# *MiRNA* Genes Constitute New Targets for Microsatellite Instability in Colorectal Cancer

**DOI:** 10.1371/journal.pone.0031862

**Published:** 2012-02-14

**Authors:** Nizar El-Murr, Zoulira Abidi, Kristell Wanherdrick, Magali Svrcek, Marie-Pierre Gaub, Jean-François Fléjou, Richard Hamelin, Alex Duval, Thécla Lesuffleur

**Affiliations:** 1 INSERM, UMRS 938 – Centre de Recherche Saint-Antoine, Equipe “Instabilité des Microsatellites et Cancers”, Paris, France; 2 UPMC – Université Pierre et Marie Curie, Paris, France; 3 AP-HP, Hôpital Saint-Antoine, Service d'Anatomie et Cytologie Pathologiques, Paris, France; 4 INSERM, U682, Développement et Physiopathologie de l'Intestin et du Pancréas, Strasbourg, France; 5 AP-HP, hôpital Saint-Antoine, Tumorothèque CancerEst, Paris, France; Yonsei University College of Medicine, Republic of Korea

## Abstract

Mismatch repair-deficient colorectal cancers (CRC) display widespread instability at DNA microsatellite sequences (MSI). Although MSI has been reported to commonly occur at coding repeats, leading to alterations in the function of a number of genes encoding cancer-related proteins, nothing is known about the putative impact of this process on non-coding microRNAs. In miRbase V15, we identified very few human microRNA genes with mono- or di-nucleotide repeats (*n* = 27). A mutational analysis of these sequences in a large series of MSI CRC cell lines and primary tumors underscored instability in 15 of the 24 microRNA genes successfully studied at variable frequencies ranging from 2.5% to 100%. Following a maximum likelihood statistical method, microRNA genes were separated into two groups that differed significantly in their mutation frequencies and in their tendency to represent mutations that may or may not be under selective pressures during MSI tumoral progression. The first group included 21 genes that displayed no or few mutations in CRC. The second group contained three genes, i.e., *hsa-mir-1273c*, *hsa-mir-1303* and *hsa-mir-567*, with frequent (≥80%) and sometimes bi-allelic mutations in MSI tumors. For the only one expressed in colonic tissues, *hsa-mir-1303*, no direct link was found between the presence or not of mono- or bi-allelic alterations and the levels of mature miR expression in MSI cell lines, as determined by sequencing and quantitative PCR respectively. Overall, our results provide evidence that DNA repeats contained in human miRNA genes are relatively rare and preserved from mutations due to MSI in MMR-deficient cancer cells. Functional studies are now required to conclude whether mutated miRNAs, and especially the miR-1303, might have a role in MSI tumorigenesis.

## Introduction

During the last decade, microRNA (miRNA) genes have been extensively identified in mammals, plants and viruses. They encode short (∼22 nucleotides) single-stranded mature RNA molecules (miRs) that regulate gene expression mostly by base pairing with the 3′ UTR of target mRNA [1], [2]. In humans, it is estimated that more than one thousand miRs control the expression of about 60% of protein-coding genes. Numerous functional studies have reported the participation of miRs in various cellular processes, and subsequently, their deregulation in a number of human diseases including cancer [2], [3]. MiRNA genes can be located within introns and less frequently within exons, or intergenic regions, their expression being regulated by independent or host gene promoters [4]. Excepting miRNA genes arising entirely from the spliced out introns of host's mRNA (named mirtrons [5]), each microRNA gene produces three types of molecules that will undergo successive cleavages by two RNAses, called Drosha and Dicer, to yield the fully functional miR: a large primary transcript (pri-miR, ∼500 to 3000 bases), a hairpin-like intermediate precursor (pre-miR, ∼60 to 80 bases), and a transient miRNA duplex from which two different mature miRs are usually produced at different (major miR/minor miR*) or equivalent (miR-5p/miR-3p) amounts [5], [6]. Each maturation step relies heavily on crucial structural features that dictate a correct and reliable biogenesis of the mature miRNA. Both size and sequence variations in various regions of the miRNA hairpin (basal segment, stem, miRNA duplex and loop) can cause dysregulation of miR biogenesis and are believed to have tumorigenic consequences [7], [8], [9], [10], [11], [12].

Several genetic and epigenetic mechanisms that lead to the alteration of miR expression and function have been described in human tumors, including colorectal cancer (CRC) [13], [14], [15]. A methylation of *miRNA-34b/c* CpG islands, for instance, has been frequently observed in CRC cell lines and primary tumors [16], and an increased expression of miR-17-92 cluster has been noted in conjunction with a chromosomal instability (CIN) at the chromosome band 13q31 containing this *miRNA* locus [17]. Importantly, CIN also called MSS (microsatellite stability) characterizes the main subset of CRC (representing 80–85% of all CRCs). Microsatellite instability (MSI), on the other hand, a particularity resulting from a DNA mismatch repair (MMR) deficiency, has been reported in the remaining 15–20% of CRCs [18]. In MSI CRC, which usually does not display CIN, tumor progression is thought to result notably from the accumulation of secondary mutational events (deletion/insertion) affecting microsatellites, repeated sequences of short DNA motifs (1–6 bp), contained in cancer-related genes [19], [20]. These mutations affect target genes involved in various biological pathways such as the regulation of cell cycle and/or cell proliferation (*TGFBR2, IGF2R, TCF4, AXIN2, PTEN, RIZ*…), the regulation of apoptosis (*BAX, CASP5, BCL10, APAF1, FAS…*), or the DNA damage signalling and repair pathways (*RAD50, BLM, MSH3, MSH6, MBD4, MLH3, CHK1, ATR…*). In the majority of target genes, frame-shift mutations were observed in exonic repeats, most often mononucleotide tracts, leading to the production of truncated proteins. More rarely, somatic mutations in intronic mononucleotide repeats of MSI target genes (*MRE11* and *HSP110*) were shown to lead to aberrant splicing and to the generation of altered proteins [21], [22].

In this study, we investigated for the first time whether miRNA genes, regardless of their genomic location, might constitute new targets of MSI in CRC. All human miRNA genes containing mononucleotide (MNR) or dinucleotide repeats (DNR) (≥7 repeats) in their hairpin sequences were screened for mutations (nucleotides additions/deletions) using a large series of MMR-deficient CRC cell lines and primary tumors, as well as lymphoblastoid cell lines (LBLs) and MSS CRC controls enabling the assessment of the polymorphic status of these sequences.

## Results

### Screening for microsatellite repeats in miRNA hairpins and determination of their polymorphism in MMR-proficient cell lines

**Figure 1:**
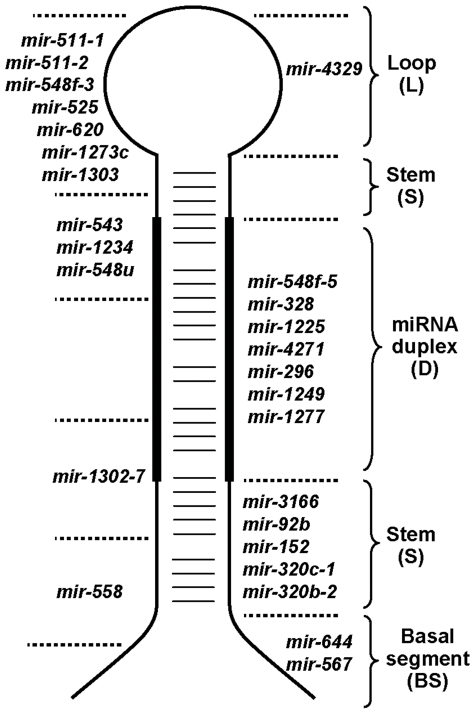
Representative scheme of miRNA hairpins with repeats spaning different locations. The basal segment (BS, single-stranded RNA), stem (S, double-stranded RNA) and terminal loop (L) are designated. The duplex (D, containing one or two potential miRs) is considered as a different entity and therefore distinguished from the stem region. Regions of the hairpin covered by MNRs or DNRs are noted for each miRNAs. To the left of the scheme are miRNA genes whose sequence repeats overlap two regions.

Amongst the 940 human miRNA sequences listed in miRbase V15, 24 contain MNR with at least seven repeat units (2.5%) (Table 1). DNR (≥7 repeats) are more rarely found in miRNA sequences (3/940, 0.3%) (Table 1). These miRNA genes are distributed on several chromosomes. The majority is found in protein-coding genes (22/27, 81.5%), mostly within intronic sequences (21/22, 95.5%). Repeated sequences vary in size and can reach up to 18 repeats. More than two-thirds of MNR (17/24; 71%) are small (7 to 8 bp) and A/T rich (16/24; 67%). They span many regions of the hairpin precursor (Figure 1) considered important for miR maturation and/or function. These regions consist of the basal segments, the stem, the miRNA duplex and the terminal loop [7], [8], [9], [10]. Two miRNAs (*hsa*-*mir-511-1* and *hsa*-*mir-511-2*) are duplicates of the same gene and display a unique sequence indistinguishable in our analysis. Except *hsa*-*mir-1234* and *hsa*-*mir-3166*, all miRNA genes were successfully amplified using a set of fluorescent primers bordering the hairpin sequence. MiRNA genes were first analyzed in healthy individuals (LBLs, *n* = 40) for an evaluation of the inherent polymorphism (Table 2). Out of 21 MNR analyzed in normal DNA samples, the majority (17 genes) was shown to be monomorphic (Table 2). Length polymorphism (1 bp shift) in *hsa-mir-1303* was observed with the smaller allele having the highest allelic frequency (Table S1, Figure S1). Single nucleotide polymorphisms (SNP) *rs33982250* and *rs34889453*, both corresponding to an Adenine deletion, are reported for *hsa-mir-1303* and are located outside the MNR [23]. Rare alleles with up to 3-bp shifts were also identified in *hsa-mir-511*, *hsa-mir-543* and *hsa-mir-1302-7* genes (Table S1, Figure S1). Similar results were obtained in a series of 25 primary MSS colorectal tumors and 13 MSS CRC cell lines (Table S1).

**Table 1:** Intergenic and intragenic microRNA genes containing MNR or DNR.

miRNA gene	Chromosomic location	Genomic location	Repeat size
hsa-mir-1302-7	8q24.3	IG	A7
hsa-mir-511-1	10p12.33	I	T7
hsa-mir-511-2	10p12.33	I	T7
hsa-mir-543	14q32.31	I	T7
hsa-mir-548f-3	5q22.1	I	T7
hsa-mir-548f-5	Xp21.1	I	T7
hsa-mir-548u	6p11.2	I	T7
hsa-mir-3166	11q14.2	IG	T7
hsa-mir-328	16q22.1	I	G7
hsa-mir-1225	16p13.3	I*	G7
hsa-mir-4271	3p21.31	E	G7
hsa-mir-92b	1q22	I	C7
hsa-mir-152	17q21.32	I	C7
hsa-mir-296	20q13.32	I	C7
hsa-mir-1249	22q13.31	I	C7
hsa-mir-320c-1	18q11.2	I	A8
hsa-mir-525	19q13.42	IG	A8
hsa-mir-320b-2	1q42.11	I	A9
hsa-mir-644	20q11.22	I	T9
hsa-mir-4329	Xq23	IG	T9
hsa-mir-1273c	6q25.2	I	T11
hsa-mir-567	3q13.2	I	A13
hsa-mir-1303	5q33.2	IG	T13
hsa-mir-1234	8q24.3	I*	G18
hsa-mir-1277	Xq24	I	(AU)7
hsa-mir-620	2q24.21	I	(UA)11
hsa-mir-558	12p22.3	I	(GU)18

IG, intergenic; I, intronic; I*, mirtron (miRNAs from small intronic sequences); E, exonic.

**Table 2:** Polymorphism and somatic mutation frequency of microsatellite repeats in miRNA genes.

			CRC cell lines			Colorectal primary tumors		
miRNA gene	LBL (%)	Polymorphism	MSS (%)	MSI (%)	P value	MSS (%)	MSI (%)	P value
**MNR**								
hsa-mir-1302-7	1/33 (3%)^a^	yes	0/13 (0%)	0/14 (0%)^b^	NS	0/24 (0%)	1/36 (2.8%)^b^	NS
hsa-mir-511	3/40 (7.5%)^a^	yes	0/13 (0%)	0/14 (0%)^b^	NS	0/23 (0%)	0/36 (0%)^b^	NS
hsa-mir-543	6/35 (17.1%)^a^	yes	0/13 (0%)	1/14 (7%)^b^	NS	0/25 (0%)	2/39 (5%)^b^	NS
hsa-mir-548f-3	0/36 (0%)	no	0/13 (0%)	0/14 (0%)	NS	0/23 (0%)	0/36 (0%)	NS
hsa-mir-548f-5	0/38 (0%)	no	0/13 (0%)	0/14 (0%)	NS	0/24 (0%)	1/40 (2.5%)	NS
hsa-mir-548u	0/32 (0%)	no	0/13 (0%)	0/12 (0%)	NS	0/23 (0%)	0/39 (0%)	NS
hsa-mir-328	0/34 (0%)	no	0/12 (0%)	0/14 (0%)	NS	0/22 (0%)	0/40 (0%)	NS
hsa-mir-1225	0/37 (0%)	no	0/13 (0%)	0/13 (0%)	NS	1/22 (4,6%)	0/33 (0%)	NS
hsa-mir-4271	0/33 (0%)	no	0/10 (0%)	0/13 (0%)	NS	0/23 (0%)	1/38 (2.6%)	NS
hsa-mir-92b	0/38 (0%)	no	0/13 (0%)	0/14 (0%)	NS	0/24 (0%)	0/40 (0%)	NS
hsa-mir-152	0/40 (0%)	no	0/13 (0%)	0/14 (0%)	NS	0/20 (0%)	0/37 (0%)	NS
hsa-mir-296	0/36 (0%)	no	0/13 (0%)	0/14 (0%)	NS	0/24 (0%)	0/38 (0%)	NS
hsa-mir-1249	0/23 (0%)	no	1/13 (7.7%)	0/13 (0%)	NS	0/24 (0%)	2/39 (5%)	NS
hsa-mir-320c-1	0/38 (0%)	no	0/13 (0%)	0/14 (0%)	NS	0/23 (0%)	1/38 (2.6%)	NS
hsa-mir-525	0/38 (0%)	no	0/13 (0%)	2/14 (14.3%)	NS	0/21 (0%)	0/33 (0%)	NS
hsa-mir-320b-2	0/37 (0%)	no	0/13 (0%)	1/14 (7.1%)	NS	0/24 (0%)	3/40 (7.5%)	NS
hsa-mir-644	0/40 (0%)	no	0/12 (0%)	6/14 (42.9%)	0.03	0/23 (0%)	8/39 (20.5%)	0.053
hsa-mir-4329	0/40 (0%)	no	2/12 (16.7%)	0/13 (0%)	NS	0/22 (0%)	6/38 (15.8%)	NS
hsa-mir-1273c	0/32 (0%)	no	0/13 (0%)	9/11 (82%)	<0.0001	0/25 (0%)	33/39 (84.6%)	<0.0001
hsa-mir-567	0/39 (0%)	no	0/12 (0%)	14/14 (100%)	<0.0001	2/25 (8%)	31/39 (79.5%)	<0.0001
hsa-mir-1303	20/39 (51%)^a^	yes	0/13 (0%)	12/14 (85.7%)^b^	<0.0001	0/23 (0%)	33/40 (82.5%)^b^	<0.0001
**DNR**								
hsa-mir-1277	0/40 (0%)	no	0/13 (0%)	0/14 (0%)	NS	0/25 (0%)	1/41 (2.4%)	NS
hsa-mir-620	32/40 (80%)^a^	yes	0/13 (0%)	1/14 (7%)^b^	NS	0/21 (0%)	1/38 (2.6%)^b^	NS
hsa-mir-558	35/40 (87.5%)^a^	yes	0/12 (0%)	4/13 (30.8%)^b^	0.095	0/24 (0%)	12/38 (31.6%)^b^	0.006

NS, not significant;

a the polymorphism rate is the percentage of normal samples showing length variations when compared to the major peak (see Table S1, Figure S1);

b mutation rates were estimated by taking into account sizes that diverge from the normal polymorphism (refer to Figure S1).

MiRNA genes with DNR appeared to be highly polymorphic (Table 2). Two of 3 genes displayed several alleles with important length variations: 6 and 15 alleles found respectively for *hsa-mir-620* and *hsa-mir-558* in healthy individuals (Table S1, Figure S1). Here, length polymorphisms have been reported to be localized within the microsatellite repeats [23].

### Mutation analysis of miRNA genes with MNR and DNR

The instability of all miRNA repeats was investigated in a series of 41 primary MSI CRCs and 14 MSI CRC cell lines (Table 2). Primary MSS CRCs (*n* = 25) and MSS CRC cell lines (*n* = 13) are used as controls. With very few exceptions, the MSS controls did not show size alterations in any of the miRNA repeats (3/557 and 3/304 mutational events in primary tumors and cell lines, respectively), while 135/913 and 50/326 mutational events were observed in MSI primary tumors (*p* = 2.2×10^−16^) and cell lines (*p* = 2.28×10^−10^), respectively. Analysis of DNR showed that 2 miRNAs with 7 (*hsa-mir-1277*) and 11 repeats (*hsa-mir-620*) were unaltered in MSI samples. *Hsa-mir-558* with 18 repeats appeared mutated in one third of MSI cell lines (4/13; 30.8%) and MSI CRCs (12/38; 31.6%) (Table 2).

**Figure 2:**
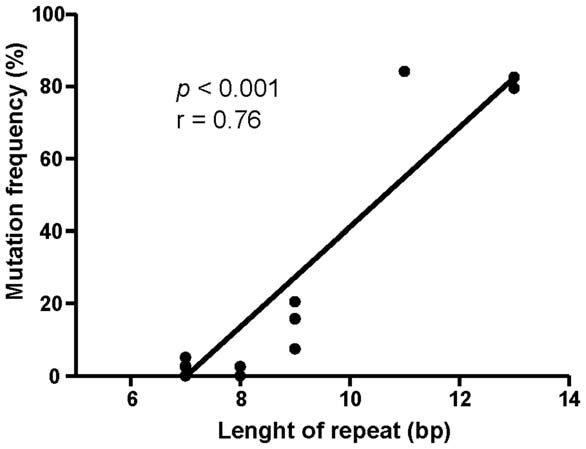
Correlation between the lengths of mononucleotide repeats in miRNAs and their mutation rates in MSI CRCs. Note the highly significant correlation observed.

Statistical studies were performed on miRNAs with MNR because of their largest number (*n* = 21). Based on the data reported in Table 2, we show that the majority of miRNA genes are rarely (3/21 with mutation rate ≤15%) or not altered (14/21, 66%) in MSI CRC cell lines, while very few genes (4/21; 19%) are found to be significantly mutated. Similar results were obtained in MSI CRCs (Table 2). However, alterations were less frequently encountered in MSI tumors compared to cell lines, an observation in accordance with what is reported for genes with coding MNRs [24]. Additionally, we noted a significant correlation between the size of miRNA MNRs and the mutation frequency in MSI tumors (*p<0.001*, r = 0.76) (Figure 2). This correlation was already observed for many microsatellite sequences independently of their genomic localizations (intergenic, coding/exonic, coding/5′ 3′ untranslated and intronic MNRs) (Figure S2).

### miRNA genes with MNR segregate into different groups

**Figure 3:**
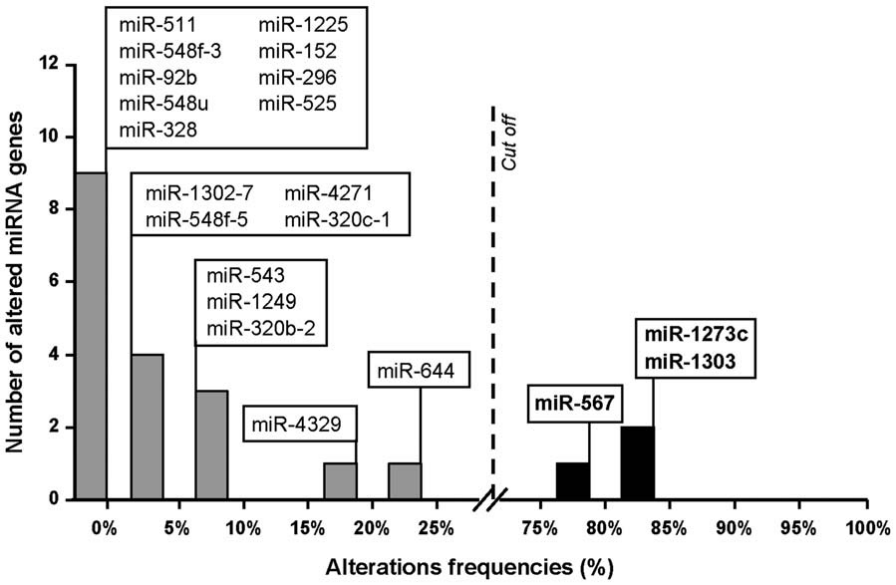
Classification of miRNAs with MNR according to their mutation frequencies in MSI CRCs. Two distinct groups of miRNAs with MNR are established based on their mutation frequencies in MSI primary tumors. The cut-off value is calculated by the ratio of likelihood statistical method and is marked by a dashed vertical line. Note that *hsa-mir-644* is included in the group of miRNAs rarely or not mutated in MSI CRCs (*n* = 18, frequency of mutation <25%) whereas *hsa-mir-1273c*, *hsa-mir-567* and *hsa-mir-1303* constitute the group of miRNAs frequently altered (*n* = 3, frequency of mutation >75%).

Using a maximum likelihood statistical method as previously described (refer to Materials and Methods and [24]), we identified two distinct groups of miRNA genes containing MNR that differed in their mutability in MSI primary tumors (Figure 3). Briefly, the test considers that all genes belong to one group of frequency and opposes to this configuration the alternative hypothesis of two mutually exclusive groups of frequencies. The likelihood ratio calculates the chances of each hypothesis and gives the most “likely” to occur. The first, largest group comprises miRNA genes found to be not or not much mutated in MSI tumors (18/21; 86%). The second group contained 3 miRNA genes (*hsa-mir-1273c*, *hsa-mir-567* and *hsa-mir-1303*) frequently mutated in MSI CRCs (≥75%). Similar groups were defined in MSI CRC cell lines (Figure S3).

### Characterization of alterations and their impact on mature miRNA expression

**Figure 4:**
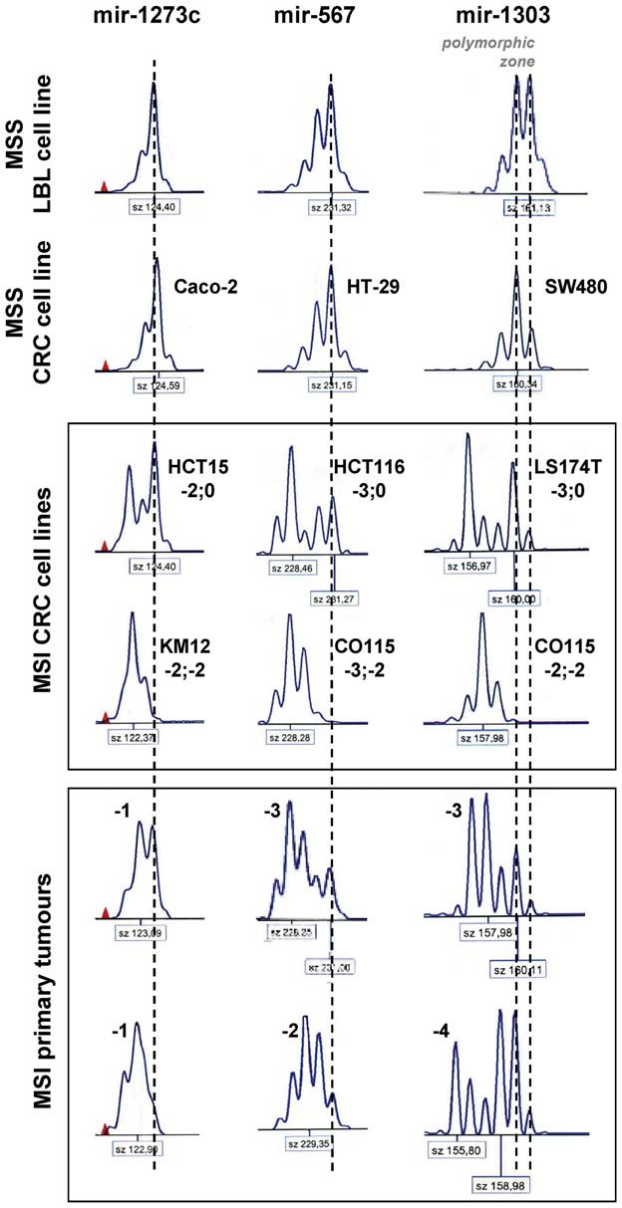
MNR instabilities in *hsa-mir-1273c* (T11), *hsa-mir-567* (A13) and *hsa-mir-1303* (T13). Allelic profiles for several MSI CRC cell lines and primary tumors are shown. Normal profiles are defined in LBL and MSS cell lines and primary tumors. For monomorphic genes, a dashed vertical line indicates the unique allele. The polymorphic zone for *hsa-mir-1303* is defined between two dashed vertical lines going along the 2 alleles (see Figure S1). Sizes (bp) are indicated in a box below each profile. Various allelic deletions ranging from 1 to 4 bp were observed in MSI CRC cell lines and primary tumors and are indicated in bold. The observed deletions were sometimes bi-allelic in MSI CRC cell lines. In MSI primary tumors, the allelic profiles were also highly suggestive of bi-allelic mutations. Due to the inherent polymorphism that can modify the length of the sequence, the hairpin sequence of *hsa-mir-1303* was determined for a correct and reliable evaluation of the alterations in MSI CRC cell lines (see Table S2).

**Figure 5:**
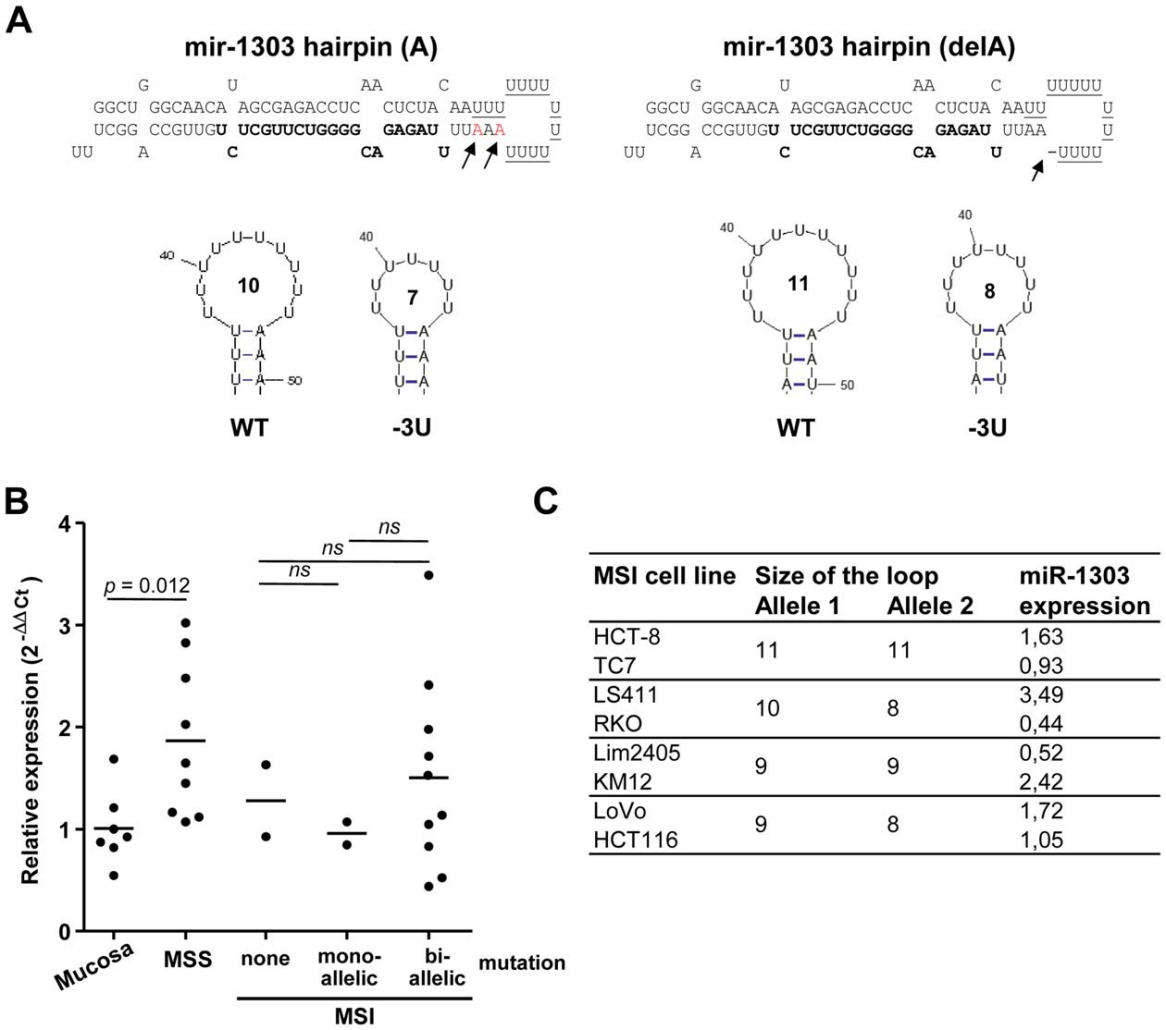
Secondary structures of WT and mutated *hsa-mir-1303* and expression levels of miR-1303 in CRC cell lines. A: Alterations in repeat sequences of *hsa-mir-1303* (A) and its variant (delA) did not seem to affect overall the secondary structure of the hairpin but the dimension of the loop (annoted inside) is slightly reduced as determined by mfold software (http://mfold.rna.albany.edu/). Mature miR (bold letters) and MNR (underlined letters) are shown in both hairpin sequences. The arrows indicate the potential positions of an Adenine deletion that leads to an enlargement of the loop. B: Comparison of the relative expressions of mature miR-1303 in MSS (unaltered MNR) and MSI CRC cell lines with none, mono- or bi-allelic mutations of *hsa-mir-1303*. MiR expression was normalized to the expression of RNU48. Means are shown for each group (black horizontal line). A significant increase in the expression of miR-1303 was observed between MSS cell lines and normal colonic mucosae (*p* = 0.012). C: Absence of correlation between the size of mir-1303 loop and the levels of mature miR-1303 expression in MSI cell lines with no (HCT-8, TC7) or bi-allelic mutations (LS411, RKO, LIM2405, KM12, LoVo, HCT116) in MNR of *hsa-mir-1303*. Note cell lines that produce hairpin precursors with the same size of the loop do express mature miR-1303 at various levels.

Our analysis allowed the identification of 3 MSI-targeted miRNAs: *hsa-mir-1273c, hsa-mir-567 and hsa-mir-1303*. For these genes, the great majority of MSI cell lines presented up to 3 bp deletions (Figure 4, Table S2), and rarely a nucleotide addition (Table S2). A bi-allelic mutation was also noted in 36%, 57% and 83% of altered MSI CRC cell lines for mir-1273c, mir-567 or mir-1303, respectively (Figure 4, Table S2). Similar alterations were found in MSI primary tumors with the exception of *hsa-mir-1273c*, which generally displayed smaller levels of alterations in tumors (only 1 bp-deletion) (Figure 4, Table S2). Concerning the polymorphic *hsa-mir-1303* gene, whose major allele displays an A-deletion (Table S1), the hairpin precursor was sequenced in each MSI CRC cell line to determine the real extent of the deletion. Like LBL and MSS cell lines (Table S1), MSI cell lines appeared to be homozygous (50%; delA/delA) or heterozygous (50%, delA/A) for the “delA” allele. The relevance of the “delA” SNP in *hsa-mir-1303* is shown in Figure 5A that illustrates changes in the terminal loop of the mir-1303 hairpin structures. The dimension of the loop containing the microsatellite decreases when the alterations get bigger and whenever the hairpin structure contains the A-addition SNP (Figure 5A).

Based on these results, we hypothesized that nucleotide deletions encountered in miRNA precursors might affect the biogenesis of mature miRs, modifying their levels of expression and/or their sequence as reported for some other miRNA genes [7], [8], [9], [10]. Using specific quantitative RT-PCR technologies, we determined the relative expression of each mature miR in wild-type (WT) and mutated CRC cell lines in comparison to expression in healthy colonic mucosae. Expression of miR-567 and miR-1273c was not detectable in colonic mucosae and colorectal cell lines (C_T_>36 cycles) (data not shown); whereas miR-1303 appeared to be fairly expressed in both normal and tumor colonic cells (Figure 5B). Taking into account the level of instability, miR-1303 expression was first analysed in MSI CRC cell lines. With normal colonic mucosae serving as controls, no difference in miR-1303 expression was noted between unaltered *hsa-mir-1303* MSI cell lines and the heterozygously mutated MSI cell lines (Figure 5B). An increase in the expression of miR-1303 expression was nevertheless observed in some of the MSI cell lines mutated on both alleles (Figure 5B). Furthermore, cell lines supposed to have identical structures of miRNA hairpins (Figure 5C), did not show comparable expression levels. It seems, therefore, as long as the size of the loop does not correlate to the levels of expression, that MSI alterations have no repercussion on mature miR expression. A significant increase of miR-1303 observed in MSS CRC cell lines in comparison to normal colonic mucosa supported this result (Figure 5B).

## Discussion

To date, the only established indirect impact of MSI process on miRNA biogenesis is the targeting and therefore disruption of protein coding genes involved in miRNA processing and transport [25], [26], [27], [28]. Our study is the first reporting somatic mutations in miRNA genes due to MSI in MMR-deficient CRCs. By screening the quasi totality of MNR and DNR (≥7 repeat units) contained in miRbase-V15-annotated miRNA hairpin sequences, *hsa-mir-1273c, hsa-mir-1303 and hsa-mir-567* were demonstrated to be mutated at high frequency in both CRC cell lines and primary tumors. Since high mutation frequency is the first criterion currently taken into consideration to identify target genes that play a role in the MSI-driven pathway to cancer [19], [20], [29], [30], these miRNA genes may thus constitute real target for MSI. In contrast, all other miRNA alterations we identified are likely to be the result of the background of genetic instability characterizing these tumors. They were found to be affected at low frequency and may play only a minor role in colon tumorigenesis, if any. Besides, some miRNA genes containing microsatellite were found to be never mutated in CRC cell lines and primary tumours. They might be considered as ‘survivor’ miRNA genes whose mutations are not selected for during tumor progression since they could be highly deleterious for cancer cells [19].

MSI is also influenced by sequence criteria, *e.g.* the length of the repeat. Expectedly, we observed an overall positive correlation between the length of miRNA repeats and their mutation rates in MSI CRC, corroborating the observation made for coding and non-coding MNR. As required for protein coding genes [29], [30], functional criteria are necessary to assert that MSI-targeted miRNA genes have a role in MSI colon tumorigenesis. MiRNAs are deeply involved in the regulation of gene expression, being therefore associated with various biological processes. No biological role has yet been attributed to *hsa-mir-1303* that is, amongst these, the only miR that was significantly expressed in the colonic mucosa. Several hundreds of mRNA can be assigned *in silico* to miR-1303 depending on the software used, and further analyses are necessary to define those whose *in vivo* expression really depends on *hsa-mir-1303*. Furthermore, the priority is to prove that MNR alterations due to MSI may impact mir-1303 function. Several teams have already highlighted the major role of the terminal loop within the primary miRNA hairpin in miRNA biogenesis and function [8], [9]. In addition, a single base alteration (i.e. SNP) within the miRNA gene itself (pri-, pre- and mature miRNA sequences) is sometimes sufficient to alter miRNA expression and/or function in cancers, blocking the processing of pri-miRNA to pre-miRNA [23], [31] or, conversely, increasing mature miR expression [32]. We failed here to observe any evident correlation between expression level of mature miR-1303 and mutation in the DNA repeat contained in its terminal loop in MSI CRC cells, regardless of the SNP adjacent to the loop. Further studies using appropriate plasmid constructions will be developed to know whether MNR mutations affecting this miRNA gene could modify the processing of the mature miR-1303 generating different miRs [33] or the function of the pri- or pre-miRNA molecules that are recently reported by Trujillo *et al.* [34] to be biologically active.

In conclusion, our findings have two main implications for the role of miRNA in MSI-driven carcinogenesis. They first provide evidence that MSI CRCs display only a few somatic mutations affecting a small number of miRNA genes containing DNA repeats with yet unclear consequence on the processing and function of these molecules. Functional studies are now required to enforce the idea that the miR-1303 might have a role in MSI tumorigenesis. Secondly, they show that DNA repeats contained in miRNA genes are relatively rare and usually preserved from mutations due to MSI in MMR-deficient cancer cells. This study focuses on the nucleotide repeats located in the hairpin sequences that contain at least the pre-miRNA, and could be enlarged to MNR located in other regions of miRNA genes important for the transcription and the processing of large primary miRNA transcripts (pri-miRNAs).

## Materials and Methods

### DNA samples

Normal DNA was obtained from 40 healthy individuals (lymphoblastoid cell lines) provided by CEPH (Centre d'Etude du Polymorphisme Humain, Paris, France). Primary tumor tissues (41 MSI and 25 MSS tumors) from patients undergoing surgery for CRC at either the Saint-Antoine hospital (Paris, France) or the hospital of Hautepierre (Strasbourg, France) were collected at the Biological Resources Centers of each institution. Written informed consent was obtained from all patients. Ethics approval was obtained from the Human Research Ethics Committee (Paris, France) and from the “Comité pour la Protection des Personnes de Strasbourg” (CPPEST IV, Strasbourg, France). The MSI status was determined by fluorescent multiplex PCR, as previously described [35], and allowed the evaluation of the percentage of epithelial carcinoma tissue in MSI samples. Only tumors with at least 40% of tumor material have been included in our study. DNA was extracted from 14 MSI and 13 MSS CRC cell lines using QIAamp DNA mini Kit (Qiagen, Courtaboeuf, France) according to manufacturer's instructions.

### Identification of mono- and dinucleotide repeats

A systematic screening for mono and dinucleotide repeats was performed in miRbase (version 15, April 2010 release) (http://www.mirbase.org/) [6]. This database provides sequences of miRNA hairpin precursors and mature miRNAs in various species. We selected human miRNAs having at least 7 repeat units. This minimum number was chosen because microsatellites were rarely found to be unstable below 7 repeats (refer to SelTarbase, http://www.seltarbase.org/).

### Mutation analysis

Specific primers flanking the hairpin sequences were designed using the Amplifix software for each miRNA candidate. PCR amplification was performed in a final volume of 25 µl with 5 ng of DNA, high or low MgCl_2_ concentration, with or without Q solution, and Taq DNA polymerase (Qiagen). Primer sequences are listed in Table S3. The thermal cycling conditions comprised an initial denaturation step at 94°C for 3 min, followed by 35 cycles at 94°C for 45 sec; 60°C for 60 sec and 72°C for 60 sec. Finnzyme Phusion High-Fidelity DNA polymerase (Thermo Fisher Scientific, Illkirch, France) was also used in some cases according to manufacturer's protocol. Adequate dilutions of the fluorescent PCR products were mixed with formamide and GeneScan™ 400HD ROX™ Size Standard (Life Technologies, Courtaboeuf, France), heat-denatured and run on a short capillary containing GS Performance Optimized Polymer 7 on the ABI 3100 Genetic Analyzer. Data were visualized and annotated in the GeneMapper 3.7 software (Life Technologies).

### Sequencing of hsa-mir-1303 hairpin precursor

A sequence containing the hsa-mir-1303 hairpin precursor was amplified by the Finnzyme Phusion High-Fidelity DNA polymerase (Thermo Fisher Scientific) using the following primers: F-GTGAACTAAACGCTGCCTCTGCTA and R-TGCAGGAACCGTACTAAGCACT (Tm = 66°C). PCR products were then purified on 96-well Multiscreen-PCR filtration plats (Millipore, Molsheim, France). Sequencing reaction was carried out using the Big Dye Terminator Kit V3.1 (Life Technologies) according to the manufacturer's protocol and using separately the forward or reverse primers. Sequences products were then purified on 96-well Multiscreen-DV plates (Millipore) with Sephadex G-50 Fine and analysed on an ABI 3100 Genetic Analyzer (Life Technologies)

### Reverse-Transcription and Real-Time quantitative PCR

Total RNA was prepared from exponentially grown cells (9 MSS and 14 MSI CRC cells) and normal colonic mucosa from patients with CRC (*n* = 7) using TRIzol reagent (Life Technologies) then quantified using a NanoDrop spectrophotometer. cDNAs were generated from 10 ng or 100 ng of total RNA using miRNA-specific stem loop RT primers (Life Technologies) for miR-1303 or miR-1273c, respectively. RT-qPCR assays were performed in triplicate on an ABI 7900 Sequence Detection System using the TaqMan MicroRNA assay according to the manufacturer's instructions (Life Technologies). For the detection of miR-567, the miScript PCR system (miScript RT and miScript SYBR Green PCR kits) was used according to the manufacturer's instructions (Qiagen). With both technologies, CT, the cycle number at which the amount of amplified target reaches a fixed threshold, was determined. CT above 36 were considered as false positives. Mature miRNA expression was normalized to that of RNU48 (Life Technologies) or RNU6B (Qiagen) (ΔCT = CT_miR_−CT_RNU_). Comparative quantification was performed using a calibrator sample. Relative miRNA expression was expressed as 2^−ΔΔCT^ (2^−(ΔCT sample−ΔCTcalibrator)^). The thermal cycling conditions comprised 45 cycles at 95°C for 15 s and 60°C for 1 min (Life Technologies) or 45 cycles at 94°C for 15 s, 55°C for 30 s and 70°C for 30 s, preceded by an initial activation step at 95°C for 15 min (Qiagen).

### Statistical analyses

The differences between variables were assessed with the *Chi-2* or Fisher's exact test, when required. Student t-test was used to evaluate differences in miR expression levels.

As previously described by Duval *et al.*, a ratio of likelihood was calculated to assign each miRNA to a frequency group [24]. With *N_i_*, the number of tumors tested at locus *i* and *n_i_*, the number of tumors unstable at this locus, the *H1* alternative hypothesis assumes the presence of two types of loci (ie. miRNA genes) that differ in their mutation frequencies (a “stable” group with a low mutation rate, *p_1_*; and an “unstable” group with a high mutation rate, *p_2_*. α and 1-α are the proportions of sites with the *p_1_* and *p_2_* instability respectively). Conversely, the *H0* null hypothesis assumes that all miRNA genes can be grouped in one frequency class, *p_0_*, where no significant differences exist between each miRNA locus. The ratio of likelihood is given by: 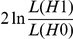


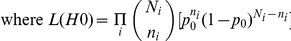


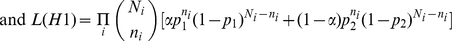



This ratio follows a Chi-square distribution with two degrees of freedom.

For all tests, a 95% confidence interval was applied and *P*<0.05 was considered as significant.

## Supporting Information

Figure S1 **Allelic profiles of polymorphic miRNA genes in LBLs.** For *hsa-mir-1303* (T_13_), *hsa-mir-620* ((TA)_11_) and *hsa-mir-558* ((GT)_18_) genes, the polymorphic zone is determined between the smallest and the largest alleles (located between the two dashed vertical lines) observed in a large series of 40 lymphoblastoid cell lines from healthy individuals. The length of the predominant alleles (bp) is indicated in a box below each profile.
(TIF)

Figure S2 **Comparison of mutation frequencies of miRNA MNRs to those of exonic, untranslated, intronic or intergenic MNRs.** MNRs with sizes between 7 and 13 bp and different genomic locations were included in this comparison. These MNRs are taken from SelTarbase (http://www.seltarbase.org/, October 2010 release), an open database of human mononucleotidic microsatellite mutations in MSI cancers.
(TIF)

Figure S3 **Classification of miRNAs with MNR based on to their mutation frequencies in MSI CRC cell lines.** Two distinct groups of miRNAs with MNR are determined according to the frequency of mutation in MSI cell lines. The cut-off value is calculated by the ratio of likelihood method and is signalled by a dashed vertical line. Note that *hsa-mir-644* is incorporated in the group of miRNAs frequently altered, that also includes *hsa-mir-1273c*, *hsa-mir-567* and *hsa-mir-1303* (*n* = 4, frequency of mutation >45%). All the other miRNAs constitute the group of miRNAs rarely altered or not altered at all (*n* = 17, frequency of mutation <15%).
(TIF)

Table S1 **Allelic distribution of polymorphic miRNA genes in LBLs and MSS colorectal tumors and cell lines.**
(DOC)

Table S2 **Size alterations of miRNA loci in MSI CRC cell lines.**
(DOC)

Table S3 **Primers sequences for mutation analysis of miRNAs.**
(DOC)
