# Supplementary figures and images for: MiRNA Genes Constitute New Targets for Microsatellite Instability in Colorectal Cancer
Source: PLoS One. 2012 Feb 14;7(2):e31862. doi: 10.1371/journal.pone.0031862 (PMC3279428; doi:10.1371/journal.pone.0031862)

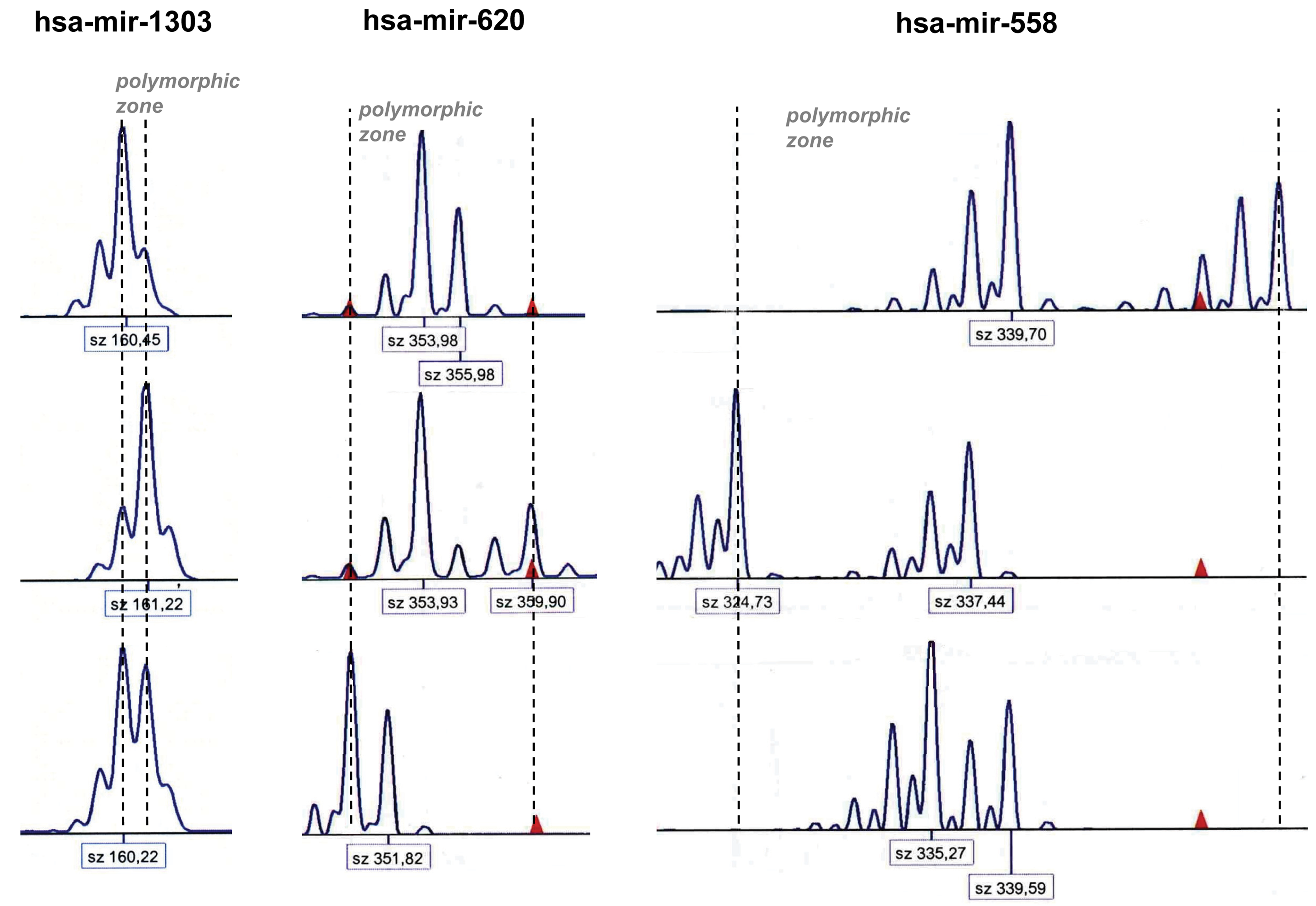

Supplement: Figure S1 — Allelic profiles of polymorphic miRNA genes in LBLs. For hsa-mir-1303 (T13), hsa-mir-620 ((TA)11) and hsa-mir-558 ((GT)18) genes, the polymorphic zone is determined between the smallest and the largest alleles (located between the two dashed vertical lines) observed in a large series of 40 lymphoblastoid cell lines from healthy individuals. The length of the predominant alleles (bp) is indicated in a box below each profile. (TIF) [file pone.0031862.s001.tif]

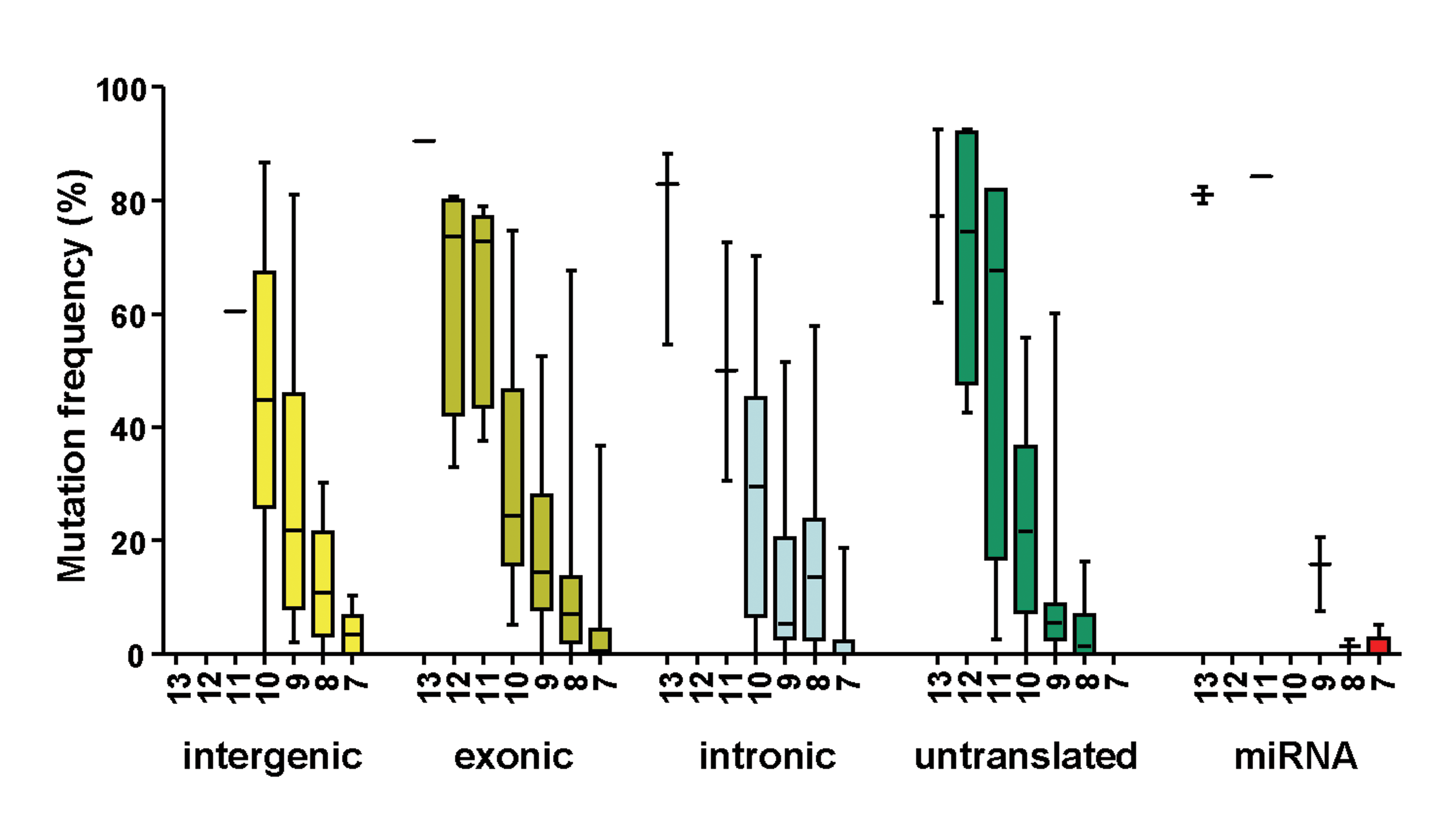

Supplement: Figure S2 — Comparison of mutation frequencies of miRNA MNRs to those of exonic, untranslated, intronic or intergenic MNRs. MNRs with sizes between 7 and 13 bp and different genomic locations were included in this comparison. These MNRs are taken from SelTarbase (http://www.seltarbase.org/, October 2010 release), an open database of human mononucleotidic microsatellite mutations in MSI cancers. (TIF) [file pone.0031862.s002.tif]

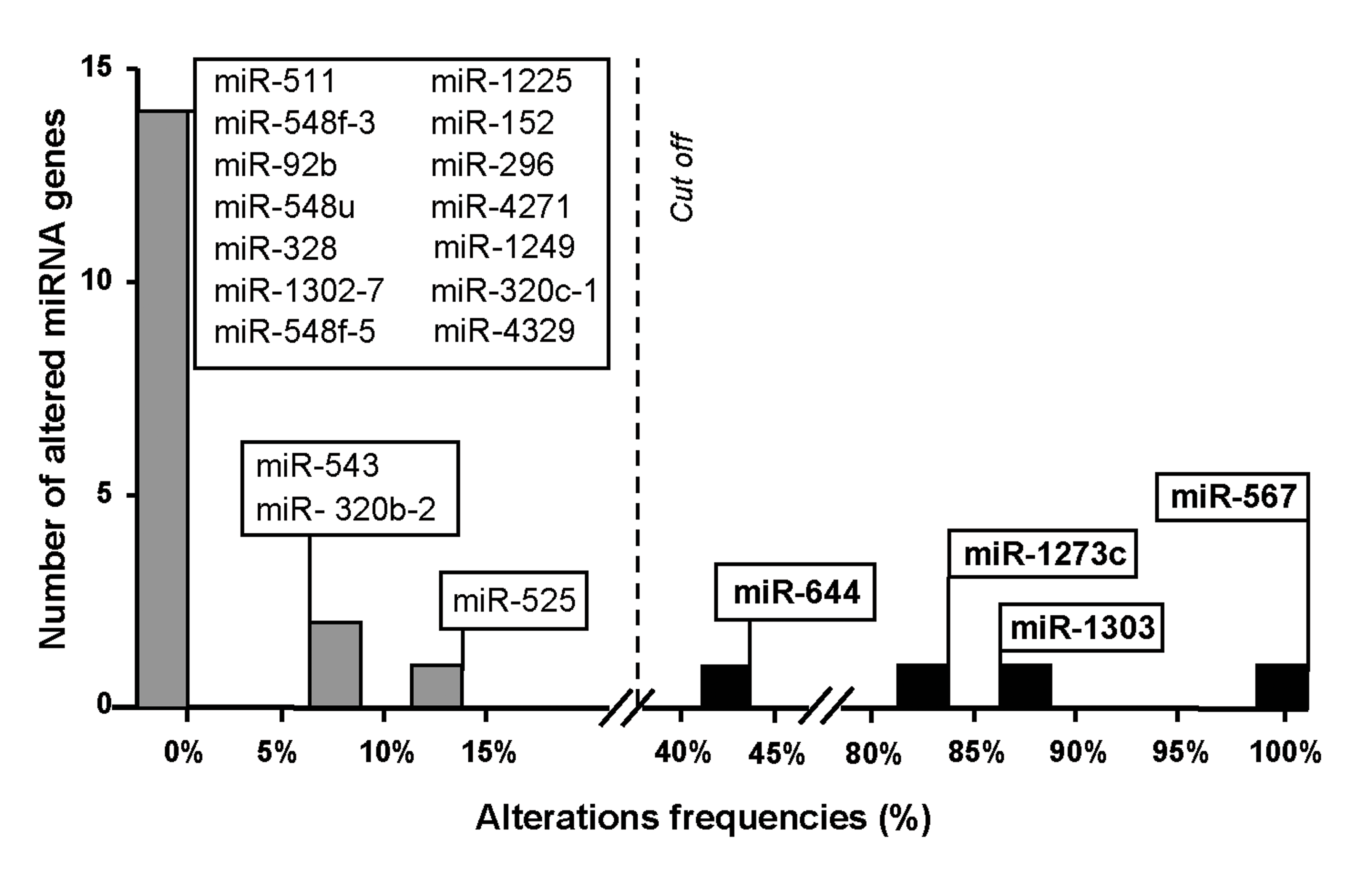

Supplement: Figure S3 — Classification of miRNAs with MNR based on to their mutation frequencies in MSI CRC cell lines. Two distinct groups of miRNAs with MNR are determined according to the frequency of mutation in MSI cell lines. The cut-off value is calculated by the ratio of likelihood method and is signalled by a dashed vertical line. Note that hsa-mir-644 is incorporated in the group of miRNAs frequently altered, that also includes hsa-mir-1273c, hsa-mir-567 and hsa-mir-1303 (n = 4, frequency of mutation >45%). All the other miRNAs constitute the group of miRNAs rarely altered or not altered at all (n = 17, frequency of mutation <15%). (TIF) [file pone.0031862.s003.tif]
