# Supplementary material for: MiRNA Genes Constitute New Targets for Microsatellite Instability in Colorectal Cancer
Source: PLoS One. 2012 Feb 14;7(2):e31862. doi: 10.1371/journal.pone.0031862 (PMC3279428; doi:10.1371/journal.pone.0031862)
Supplement: Table S1 — Allelic distribution of polymorphic miRNA genes in LBLs and MSS colorectal tumors and cell lines. (DOC) [file pone.0031862.s004.doc]

**Table S1.** Allelic distribution of polymorphic miRNA genes in LBLs and MSS colorectal tumors and cell lines

| **miRNA gene** | **Alleles (bp)** | **LBL (%)** | **Cell lines (%)** | **Primary tumors (%)** |
| --- | --- | --- | --- | --- |
| hsa-mir-1302-7 | a (285 bp) | 32/33 (97%) | 13/13 (100%) | 24/24 (100%) |
|  | b (282 bp) | 0/33 (0%) | 0/13 (0%) | 0/24 (0%) |
|  | a/b | 1/33 (3%) | 0/13 (0%) | 0/24 (0%) |
| hsa-mir-511 | a (195 bp) | 37/40 (93%) | 13/13 (100%) | 19/23 (83%) |
|  | b (194 bp) | 3/40 (7%) | 0/13 (0%) | 4/23 (17%) |
|  | a/b | 0/40 (0%) | 0/13 (0%) | 0/23 (0%) |
| hsa-mir-543 | a (218 bp) | 29/35 (83%) | 13/13 (100%) | 25/25 (100%) |
|  | b (219 bp) | 0/35 (0%) | 0/13 (0%) | 0/25 (0%) |
|  | a/b | 6/35 (17%) | 0/13 (0%) | 0/25 (0%) |
| hsa-mir-1303* | a (160 bp) | 19/39 (49%) | 6/13 (46%) | 10/23 (43%) |
|  | b (161 bp) | 4/39 (10%) | 2/13 (15%) | 3/23 (13%) |
|  | a/b | 16/39 (41%) | 5/13 (39%) | 10/23 (43%) |
| hsa-mir-620* | a (356 bp) | 8/40 (20%) | 3/13 (23%) | 2/21 (10%) |
|  | b to f | 32/40 (80%) | 10/13 (77%) | 19/21(90%) |
| hsa-mir-558* | a (337 bp) | 5/40 (12%) | 4/12 (33%) | 2/24 (8%) |
|  | b to j | 35/40 (88%) | 8/12 (67%) | 22/24 (92%) |

The letter “a” is assigned to the major, most abundant alleles; letters “b” to “j” are assigned to the minor, more or less rare shifted forms of the amplicon; *, miRNA genes reported as being polymorphic in Patrocles miRNA database ([http://www.patrocles.org](http://www.patrocles.org/)) and by Duan et al. [23]. On the basis of the percentage obtained for *hsa-mir-1303*, the major allele “a” displays an A-deletion (delA).
