# Supplementary material for: MiRNA Genes Constitute New Targets for Microsatellite Instability in Colorectal Cancer
Source: PLoS One. 2012 Feb 14;7(2):e31862. doi: 10.1371/journal.pone.0031862 (PMC3279428; doi:10.1371/journal.pone.0031862)
Supplement: Table S2 — Size alterations of miRNA loci in MSI CRC cell lines. (DOC) [file pone.0031862.s005.doc]

**Table S2.** Size alterations of miRNA loci in MSI CRC cell lines

| **Cell lines** | **mir-1273c** | **mir-567** | **mir-1303** |
| --- | --- | --- | --- |
| Co115 | -1 ; -1 | -3 ; -2 | -3 ; -2 |
| HCT116 | -1 ; 0 | -3 ; 0 | -3 ; -2 |
| HCT15 | 0 ; -2 | 0 ; +1 | -1 ; 0 |
| HCT8 | 0 ; 0 | 0 ; +1 | 0 ; 0 |
| KM12 | -2 ; -2 | -1 ; 0 | -3 ; -1 |
| LIM1215 | 0 ; 0 | -3 ; -1 | -3 ; -1 |
| LIM2405 | -1 ; +1 | -2 ; 0 | -2 ; -2 |
| LoVo | -1 ; 0 | -2 ; -1 | -3 ; -2 |
| LS174T | -1 ; 0 | -2 ; 0 | -3 ; -1 |
| LS411 | -1 ; -3 | -2 ; -2 | -2 ; -1 |
| RKO | -1 ; 0 | -2 ; -1 | -2 ; -1 |
| SW48 | -1 ; 0 | -2 ; -1 | -3 ; 0 |
| TC71 | -1 ; 0 | -2 ; -1 | -2 ; -1 |
| TC7 | 0 ; 0 | -3 ; -2 | 0 ; 0 |

“-“ or “+” represent nucleotide deletions or additions, respectively.

Following numbers characterize the extent of the deletion or addition. Sequencing of *hsa-mir-1303* hairpin sequence was performed, and accordingly many cell lines having the “A” allele displayed a more extended deletion contrary to what was initially thought by genotyping (refer to Figure 4, LS174T and Co115).

Note that the alterations observed are bi-allelic in more than half of MSI CRC cell lines for mir-567 and mir-1303.
