# Supplementary material for: MiRNA Genes Constitute New Targets for Microsatellite Instability in Colorectal Cancer
Source: PLoS One. 2012 Feb 14;7(2):e31862. doi: 10.1371/journal.pone.0031862 (PMC3279428; doi:10.1371/journal.pone.0031862)
Supplement: Table S3 — Primers sequences for mutation analysis of miRNAs. (DOC) [file pone.0031862.s006.doc]

**Table S3.** Primers sequences for mutation analysis of miRNAs

| **miRNA gene** | **5’-3’Forward and Reverse Primers** | **Amplicon size** | **Amplicon location (GRCh37.p2)** |
| --- | --- | --- | --- |
| hsa-mir-525 | CTGGAGATGGTCGTTTTAGG | 242 bp | 19: 26468859-26469100 |
|  | ACGTTGCTTTACCCAAACCG |  |  |
| hsa-mir-567 | TAGGTACTAGGTGGAAGGAG | 230 bp | 3: 18326730-18326959 |
|  | CCACTGAAAGGCAATGGAAG |  |  |
| hsa-mir-1302-7 | CTAGGTCCTGTCCATGCTC | 284 bp | 8: 56141340-56141057 |
|  | CAGGATTCCACAGATGGTGA |  |  |
| hsa-mir-320b-2 | GCTGGGTTGAGAGGGCAAAAGGAA | 237 bp | 1: 17962547-17962311 |
|  | CCAGACCTTGGAGCCATGCTTGA |  |  |
| hsa-mir-320c-1 | ATGAGGCCTTCTCTTCCCAGTTCT | 220 bp | 18: 752585-752797 |
|  | AAGGAAATTCCCTTAGTCTGTGTTCATTCA |  |  |
| hsa-mir-328 | GTCGAAGTCCTCCTGTGTAG | 182 bp | 16: 20850632-20850451 |
|  | GGGCTGTATGCACTTTCTC |  |  |
| hsa-mir-1225 | GATGGGCCTCAGCAAGGTCAA | 153 bp | 16: 2080310-2080158 |
|  | AGCGGCTCCATCCCTTCAA |  |  |
| hsa-mir-1249 | AGCTGCCAGGACTTTGGTG | 196 bp | 22: 24987574-24987379 |
|  | TCTTGAAGGACGAGTAGGAG |  |  |
| hsa-mir-152 | GGGCATGCTTCTGGAGTCTA | 174 bp | 17: 11388867-11388695 |
|  | AGTTCTGTCATGCACTGACTG |  |  |
| hsa-mir-296 | GGGAAGATCCTGAGTGAAATTG | 195 bp | 20: 27588901-27588707 |
|  | GTATCGACTGTCCTGTCTCCA |  |  |
| hsa-mir-92b | CCGCCAATATTGCACTCG | 218 bp | 1: 6653664-6653880 |
|  | GAGGTGCTGGATGGAGTTAAG |  |  |
| hsa-mir-1303 | AGATCAGGCTGGGCAACATAGCGA | 162 bp | 5: 15228257-15228418 |
|  | TCACGAGGTAGCTTGGGGAAGCA |  |  |
| hsa-mir-511-1 | GACACCCATCGTGTCTTTTGCTCT | 194 bp | 10: 273903-274096 |
|  | TTCAGCTGATGGACTTCCTTCGAG |  |  |
| hsa-mir-511-2 | GACACCCATCGTGTCTTTTGCTCT | 194 bp | 10: 17827112-17827305 |
|  | TTCAGCTGATGGACTTCCTTCGAG |  |  |
| hsa-mir-543 | CACGGCCATATCTTTGTCAC | 217 bp | 14 : 82498204-82498420 |
|  | CGTCTTCAAGGCAGAATAGG |  |  |
| hsa-mir-548f-3 | AATGCCCAGAGATTGGGAGTGCAGA | 169 bp | 5: 18163560-18163393 |
|  | ACGGCAATTGTATTAGGTTGGTGCAAAAG |  |  |
| hsa-mir-548f-5 | TGGTTTAGCGATTAGAGAAATGA | 285 bp | X: 30541628-30541344 |
|  | TTTGATGTTGCTATTAGGTTGGT |  |  |
| hsa-mir-644 | CCCCTCATTCTGTTAATGTG | 229 bp | 20: 3250087-3250315 |
|  | TAGGGAACCATCTTTGCTC |  |  |
| hsa-mir-4271 | GCAGATCCTTTGTGACCTCTTG | 292 bp | 3: 49251494-49251785 |
|  | CTGCGATGCTCGTGAGTCATTA |  |  |
| hsa-mir-4329 | GACAAGAGCTGGAAGGGGAGCCTA | 248 bp | X: 35320446-35320199 |
|  | TCTAAGCCTTGGCCTCAATCGGTA |  |  |
| hsa-mir-548u | TGTCAGGCACCAGTTCATTTGGTC | 157 bp | 6: 57194851-57195007 |
|  | AGGTTGGCGCAAAAGTAATTGCAG |  |  |
| hsa-mir-1273c | GCTTCAGTGAGCTATGTTCACG | 126 bp | 6: 59343920-59344045 |
|  | AGAGGTGAGATCATGCACTCCA |  |  |
| hsa-mir-558 | TCCTGTTTCAGGTGCCATTTCC | 320 bp | 2: 11578890-11579209 |
|  | GTTTTAAGCCAGCCCGTTTGTG |  |  |
| hsa-mir-620 | AATGAGGAGTGCTACTGCAAGTGG | 356 bp | 12: 7162739-7163094 |
|  | GCAAAGCCTCCTTTCTTAGG |  |  |
| hsa-mir-1277 | TCCAGCAGAAATGCAGTATCC | 315 bp | X: 1788039-1788353 |
|  | GATTTAAGCAAGGGGCTGAA |  |  |
